# Buffalo Medical and Surgical Association

**Published:** 1883-07

**Authors:** C. M. Daniels

**Affiliations:** Secretary


### Society Reports.

### Buffalo Medical and Surgical Association.

*Adjourned Regular Meeting, Tuesday, June 12th, 1883.*

President, Dr. John Cronyn, in the Chair. Dr. C. M. Daniels, Secretary.

Present—Drs. Bartlett, Frank, Strong, Fell, Coakley, Brecht, Granger, Hopkins, Heath, Fowler, F. H. Potter, Hartwig, Barton, Moody, Hubbell, Howe, Crego, W. W. Potter, and, by invitation, Drs. Weil, Paulding, Bartlett, Jr., L. A. Bull, Messrs. H. Poole, C. D. Zimmerman and Miss B. Barrett.

Applications for membership received from Drs. Chas. Weil and H. E. Hayd, who were duly elected.

Essay by Dr. F. W. Bartlett; subject, "The Practical Utilization of Ozone in the Treatment of Disease."

Dr. Fell presented his notes of investigation upon the "Effects of Ozone upon Micro-organisms of Infusions," giving in detail the result of microscopical examinations, etc.

*Discussion*—Dr. Hartwig expressed himself as being pleased with the able and exhaustive paper. Although personally he had not investigated the subject sufficiently to determine its value, yet, with a little experience in typhoid fever, he had not been able to bring the mortality down to ten per cent., as the essayist stated had been done. Also mentioned that ozone itself had the property of inducing sleep.

Dr. Strong acknowledged his ignorance of the subject, and thought "confession was good for the soul." Considered that we had neglected this practical point in the treatment of disease. Also expressed his unbounded admiration for the fidelity with which Dr. Bartlett had pursued his investigations. He believed there was great merit and value in ozone, if practicable, and that young men should be encouraged to carry out these views and investigate further.

Dr. Hopkins said the association should put itself on record toward Dr. Bartlett regarding his investigations. He believed that whenever one went out of the beaten path to enlarge his scope of knowledge, he would be very sure to bring in something new and valuable. As far as his observations went it was not proven that disease was always cured by the agent used; also, that the statement of a certain per cent. of fatal cases caused confusion. As the exact percentage was very difficult to determine, many things might influence each individual case.

Dr. Heath stated that to him the paper seemed hardly specific enough. He should be pleased to know just how ozone acted, either in health or disease.

Dr. Howe was much interested, and said we have been taught that we should always be on our guard against receiving statements that had not been thoroughly sifted. It often seemed that just when we were getting at the bottom of facts, we diverged, and that further investigation upon ozone was eminently desirable. Asked Dr. Fell to explain what he meant by an "ozonized atmosphere."

Dr. Fell stated it was where ozone was present in sufficient quantity to be detected by the odor, and that air be respirable.

Dr. Howe further stated that it would be very desirable to know the degree and proportion of ozone used.

Dr. Hubbell said that a few years ago he made practical use of ozone in an epidemic of malignant diphtheria, but failed to get any direct beneficial results.

Dr. Hayd was somewhat surprised that results were sometimes so negative. To us as physicians the importance lay in the practical illustrations. Thought Dr. Fell's examinations not sufficiently clear to be accepted as positive results or authority.

Mr. Poole, in referring to the point raised by Drs. Howe and Heath, said that the amount of ozone was extremely difficult to determine. It had been stated by authority that five per cent. was the largest amount obtainable, although he thought it could be more, but not more than one-thousandth of its bulk.

Dr. Cronyn wished to pay Dr. Bartlett the compliment that his labor and pains deserved, although he was somewhat of an enthusiast. In this, as in others, proof being needed, he should find it exceedingly difficult to believe that all diseases could be cured by it. As to its value as a disinfectant, he had no doubt. Its history is a long one. Believed it had been found exceedingly useful in cholera, but injurious in pulmonary diseases. Thought it of value in crowded places, as hospitals, ships, etc. Considered that the papers of Schoenbein had fallen into disuse, and that much further investigation was needed to prove its true value in disease.

Dr. Bartlett was very much obliged to the association for the kindly manner in which his paper was received. He did not present it as being strictly scientific, as he could not detail, for want of time. He had been entirely original in his reports, having had no co-operation or aids in way of mechanical ingenuity. Had been liberal in every case cited, and his percentage of mortality given was rather under than above that stated. Said we were assuming as a fact that micro-organisms do damage, although the conditions were not understood, and that at times they are evidently quite harmless. Regarding the deodorant power of ozone, he believed that where it was used in sick rooms, contagious or infectious diseases were less likely to be transmitted. Also, exhibited and explained his generator, and said the amount of ozone could only be approximated.

Dr. Frank asked what was the temperature of ozone.

Dr. Bartlett stated it was not, as yet, ascertained, although it was destroyed at 185 degrees Fahrenheit. Believed it most active at ordinary temperature of the body.

Adjourned. C. M. Daniels, *Secretary.*

